# Nurses' Indoor Uniform in the Streets: Unhygienic and Unprofessional

**Published:** 1916-08-26

**Authors:** 


### Nurses' Indoor Uniform in the Streets: Unhygienic and Unprofessional.

*To the Editor of* The Hospital.

Sir,—I was very interested in your remarks in The Hospital of August 5 *re* the wearing of ward uniform out of doors. Nurses during their training are supposed to be taught asepsis, and yet the authorities of large training-schools allow the nurses to go out of doors in public vehicles and London streets with not only the ward dress, but also an apron on! To a Guy's-trained nurse this sight in London is a positive eyesore, and I think hospital authorities and patients (for after all it is they who suffer) owe you a debt of gratitude for ventilating the matter.—Yours faithfully,

A Guy's-trained Matron.

